# The Rebooting in Sports and Physical Activities After COVID-19 Italian Lockdown: An Exploratory Study

**DOI:** 10.3389/fpsyg.2020.607233

**Published:** 2020-11-25

**Authors:** Marco Guicciardi, Riccardo Pazzona

**Affiliations:** Department of Education, Psychology, Philosophy, University of Cagliari, Cagliari, Italy

**Keywords:** COVID-19, physical activity, sports, lockdown, regulatory self-efficacy

## Abstract

The lockdown imposed in Italy to reduce the spread of COVID-19 posited unusual challenges to people practicing sports and physical activities. The rebooting of activities highlighted the need to cope with new behaviors and routines, such as wearing a face mask while exercising. We conducted a web-based survey in Italy at the start of physical activities’ rebooting, to investigate how people reacted to the new norms. Participants completed the questionnaires assessing insomnia, regulatory self-efficacy, optimism, mood states, and mental toughness. Age, gender, and environment were assumed as design variables. Results showed that in outdoor activities, the younger females as compared to the older manifest less regulatory self-efficacy, while the younger males manifest more regulatory self-efficacy than the older. In indoor activities, a reversed pattern of regulatory-self-efficacy was observed. Regarding life orientation, younger participants showed less optimism and positive expectation for the future and seem to be more exposed to the unexpected effects of the restrictions: they showed more sleep disturbances, confusion, depression, anger, and fatigue and less vigor and mental toughness than older participants. An understanding of the psychological implications of the rebooting phase can support the enactment of more appropriate behaviors to practice sports and physical activities when living at the time of the coronavirus.

## Introduction

In winter 2019, a new coronavirus (COVID-19) appeared in the world. It is an infectious respiratory disease caused by the airborne SARS-Cov2 virus. The clinical manifestations of COVID-19 are not specific, ranging from asymptomatic infection to severe respiratory failure. An infected person may experience symptoms such as fever, cough, myalgia, fatigue, and dyspnea after an incubation period that can range from about 2 to 14 days. During this time, some infected persons can be contagious. COVID-19 caused hundreds of thousands of deaths and millions of infected people, leading the World Health Organization to classify it as a pandemic in March 2020. Across the world, preventive measures have been taken to limit the spread of the virus, some more restrictive, others more permissive, depending on the nation and on the spread of the disease.

Italy has adopted a series of public health measures aimed at restraining the spread of the virus and limiting contagions, such as self-isolation and social distancing. To ensure these preemptive measures and restrict the movement of the citizens, schools, public places, and businesses were shut down. People have been forced to stay at home to mitigate the transmission of the virus. The population was allowed to go out only for reasons of primary necessity, for example, to accomplish specific activities (health visits, purchasing medicines or food) or to perform essential work (healthcare and social care operators, police and armed forces, firefighters).

The lockdown affected the life habits of people who unexpectedly have had to change behaviors, commitments, and ways of living and working (Chen et al., 2020; Favieri et al., 2020; López-Bueno et al., 2020a). Sports and physical activities were involved too: gyms, stadiums, pools, dance and fitness studios, physiotherapy centers, parks, and playgrounds were closed, compelling people to find in-home alternatives to exercise (Lesser and Nienhuis, 2020; Maugeri et al., 2020).

Many studies have documented the impact of these prolonged restrictions on the mental and physical health of Italian citizens (Conversano et al., 2020; Di Giuseppe et al., 2020; Favieri et al., 2020; Flesia et al., 2020; Moccia et al., 2020; Rossi et al., 2020; Somma et al., 2020). Most of the studies have highlighted an increase in distress, which affected principally women, younger citizens, patients already suffering from one or more chronic pathologies such as diabetes and cardiovascular disease, or people more exposed to the risk of contagion (i.e., health workers). Insomnia, anxiety, depression, confusion, anger, fear of contagion, and financial insecurity were the symptoms more frequently reported by interviewed participants (Brooks et al., 2020; Casagrande et al., 2020; Mazza et al., 2020; Rossi et al., 2020; World Health Organization [WHO], 2020), in a similar way to people suffering from post-traumatic stress disorder (PTSD) (Brooks et al., 2020; Forte et al., 2020).

During the lockdown, an animated debate has developed about the opportunity to completely prohibit outdoor physical activity or limit it in the neighboring places to home. On one side, safety procedures, such as physical distancing or using personal protective equipment (i.e., mask and gloves), should suggest limiting physical activity only to home-based exercise or to outdoor individual fitness activities as jogging, running, and biking. On the other side, an unintended consequence of the rigid application of the safety measures may be the reduction of physical activity tout court, associated with the mental and physical risks of increased sedentary behavior (Ekelund et al., 2019).

Physically active individuals generally experience less anxiety, stress, depression, and PTSD (Rosenbaum et al., 2011, 2014; Vancampfort et al., 2017; Chekroud et al., 2018). Moreover, a moderate-intensity physical activity can exert a protective role against bacterial and viral infections (Martin et al., 2009) and may enhance the immune function (Dixit, 2020), mainly in less fit subjects or sedentary population. Indeed, physical activity can also prevent some chronic diseases such as diabetes, cancer, cardiovascular diseases, and reduce their more harmful complications (Guicciardi et al., 2019a,b).

The growing scientific evidence related to COVID-19 underlines the importance of maintaining a regular physical activity even during the lockdown, regardless of the age and type of activity previously carried out (Chen et al., 2020; López-Bueno et al., 2020b). Physical activity may promote the release of stress hormones responsible for reducing excessive local inflammation within the respiratory tract. It may also induce the secretion of anti-inflammatory cytokines, modulate T-helper cell population activity, and minimize cell damage and necrosis (Ravalli and Musumeci, 2020).

Some scholars have documented the adverse effects of lockdown in Italian athletes and in the more active population (Chirico et al., 2020; Di Fronso et al., 2020; Maugeri et al., 2020). During the prolonged lockdown, the levels of physical activity of people classified before the COVID-19 as highly and moderately active drastically decreased; instead, individuals classified before the COVID-19 as low active significantly increased total weekly physical activity energy expenditure during the quarantine (Di Fronso et al., 2020). This pattern could be probably due to the greater housework activities carried out by people forced to stay at home. More active people, females and younger adults, reported more psychological discomfort associated with the reduction of levels of physical activity (Maugeri et al., 2020). Comparing the perceived stress and the psychobiosocial states of Italian athletes before and during the lockdown, Di Fronso et al. (2020) found that female and novice athletes reported scores on perceived stressors and dysfunctional psychobiosocial states higher than male and elite/expert athletes, respectively.

Using an integrated approach, Chirico et al. (2020) investigated psychological determinants of the physical activity behavior, when the first official lockdown was adopted in Italy (March 11, 2020), which had permitted about a dozen of days to perform outdoor physical activities and sports while maintaining social distancing.

In May 2020, after 2 months of lockdown, Italy, like the rest of the world, was also allowed to resume sports and physical activities. This rebooting phase was conditioned by various restrictions such as physical distancing, the use of personal protective equipment (mask and gloves), sanitizers, and the need to isolate personal equipment and clothes in closed bags. These restrictions helped to understand that the rebooting phase could be problematic as it is not merely a matter of restoring usual sport and physical activities but adapt old habits to the new regulation, also during sport or leisure activities. Sports and physical activities, regardless of the type and level at which they were previously practiced affect multiple psychological variables related to changing circumstances and habits due to COVID-19. The “new normality” requires a subtle and implicit change in everyday routines, which affects the resumption of sports and physical activities (Drury et al., 2020; Hughes et al., 2020). For example, having to train in a gym, pool, or fitness studio by sharing space with other people who may be contagious can affect the individual’s self-regulatory efficacy, anxiety, or the fear of contagion, enhanced by physical proximity.

The objective of this study is to gain an understanding of the impact of sports and physical activities rebooting in an Italian sample of adult individuals. Specifically, we aim to explore differences due to the gender, age, and environment on multiple psychological variables. Notably, we hypothesize that females, younger individuals, and individuals who practice indoor physical activities have more remarkable difficulties in rebooting of sports and physical activities than males, older individuals and those who practice outdoor physical activities. Starting from these hypotheses, we investigated these differences in sleep disturbances, regulatory self-efficacy, optimism, mood states, and mental toughness. We expected that a good rebooting of physical activities could be associated with low levels of insomnia, high levels of regulatory self-efficacy, positive moods, optimism, and mental toughness.

## Materials and Methods

### Sample

To take part in the study, participants had to be over the age of 18 and had to read and write in Italian to complete the online survey. Participants were recruited through snowball sampling using social media, email, and informal network.

The link allowing access to the survey broadcasted through forums, personal blogs, and social networks (Twitter, Whatsapp, Facebook, and Linkedin) and people that were practicing different sports and physical activities were invited to complete it. Participants were informed about the aims of the research and chose to participate voluntarily and anonymously. Data were collected from June 1 to 30, 2020. All subjects gave their informed consent for inclusion before they participated in the study. The study was conducted in accordance with the Declaration of Helsinki, and the Ethics Committee of the University of Cagliari approved the protocol (n.0161970/2020). The completion of the online survey lasted about 10 min.

Although 405 participants enrolled in the online survey, 72 questionnaires (18%) were incomplete and therefore removed from the dataset. The study consisted of the resultant participant sample of 333 active Italian adults, practicing different sports and physical activities, as soccer, basket, dance, running, and bodybuilding prevalently for at least 2 years with a frequency of two or more times a week. Of those 333, 41% were females, 61% were younger adults (under 30), and 64% were individuals practicing indoor sports and physical activities (Table 1).

**TABLE 1:** Descriptive statistic of the sample.

**Characteristics**	**Group**	***N*(%)**
Gender	Male	195 (59%)
	Female	138 (41%)
Age range	Younger (<30)	202 (61%)
	Older (≥30)	131 (39%)
Environment	Indoor	214 (64%)
	Outdoor	119 (36%)
Area	Countryside	103 (30.9%)
	Suburban area	37 (11.1%)
	Urban city	192 (57.7%)
	Missing	1 (0.3%)
Occupation	Student	161 (48.4%)
	Unemployed	15 (4%)
	Employed	149 (44.7%)
	Retired	8 (2.4%)
Sports and physical	<2 years	38 (11.4%)
activity practice	≥2 years	295 (88.6%)
Training	Once a week	17 (5.1%)
	2–3 times a week	146 (3.8%)
	More than 3 times a week	170 (51.1%)
Covid examination	Yes	38 (11.4%)
Covid positive	Yes	3 (0.9%)
Deaths among infected	Yes	19 (5.7%)
acquaintances		

### Measures

Participants completed questionnaires using an online survey software (Lime Survey) at the beginning of phase 2 (“Rebooting”), which followed the Italian lockdown lasting almost 3 months (from March to May 2020). The survey consists of two parts: the first one provided sociodemographic data (gender, age, province of residence, occupation) and some information about the physical activity performed before and during the lockdown. The second one comprised several scales now detailed.

The *Insomnia Severity Index* (ISI) is a short questionnaire developed for an easy and brief clinical assessment of insomnia severity (Bastien et al., 2001). The ISI comprises six items rated on a point Likert scale, ranging from 0 to 4 and anchored differently to the content of the item: for example, from “no problem” to “very severe” (items 1, 2, 3), from “very satisfied” to “very dissatisfied” (item 4), and from “not at all” to “very much” (items 5, 6, 7). The ISI assesses the severity of sleep onset, the severity of sleep maintenance, early morning awakenings, satisfaction level with current sleep pattern, interference with daily living, noticeability of impairment due to sleep difficulty, and level of distress caused by the sleep problem. The total score of the questionnaire is partitioned in the following categories: 0–7, no significant insomnia; 8–14, subthreshold insomnia; 15–21, moderate insomnia; and 22–28, severe insomnia. The cutoff value of 15 represents the threshold for a diagnosis of clinically relevant insomnia. The ISI is a worldwide instrument used for the assessment of insomnia; we used the Italian validated version of the ISI (Castronovo et al., 2016). In our study, the Cronbach’s alpha was 0.75.

The *Life Orientation Test-Revised* (LOT-R) (Scheier et al., 1994) assesses individual differences in generalized optimism and positive expectations for future outcomes. The LOT-R comprises three positively worded items measuring optimism (Items 1, 4, 10), three negative framed items measuring pessimism (Items 3, 7, 9), and four fillers (Items 2, 5, 6, 8). The items were measured on a five-point Likert scale ranging from 1 “strongly disagree” to 5 “strongly agree.” The scores related to negatively worded items that measured pessimism were reversed. We used the Italian validated version of LOT-R (Giannini et al., 2008). In our study, the Cronbach’s alpha was 0.78.

The *Italian Mood Scale* (ITAMS) was developed by Quartiroli et al. (2017) for use in sport and exercise to evaluate six mood states: anger, confusion, depression, fatigue, tension, and vigor. The ITAMS comprises 24 words expressing feelings. The feelings are rated on a five-point Likert scale, ranging from 0 “not at all” to “extremely.” Each mood state is assessed through six items: anger (items 7, 11, 19, 22), confusion (items 3, 9, 17, 24), depression (items 5, 6, 12, 16), fatigue (items 4, 8, 10, 21), tension (1, 13, 14, 18), and vigor (items 2, 15, 20, 23). In our study, the Cronbach’s alpha was 0.91.

The *Mental Toughness Questionnaire* (MTQ-10) assesses mental toughness and its facets: challenge, commitment, control, and confidence (Papageorgiou et al., 2018). The questionnaire comprises 10 items that assess, respectively: challenge (items 3, 6), commitment (items 2, 7), control (items 1, 8, 9), and confidence (4, 5, 10). All items are rated on a five-point Likert scale ranging from 1 “strongly disagree” to 5 “strongly agree”. The Italian version of MTQ-10 was used (L. Girelli, personal communication, May 28, 2020). In our study, the Cronbach’s alpha was 0.80.

The *Regulatory Self-Efficacy Scale for Sport Rebooting* (RSE-SR) was purposely developed by the research team, to assess the regulatory self-efficacy of athletes facing the rebooting of sports activities after lockdown. Based on Bandura’s recommendation (Bandura, 1986), RSES-SR comprises 11 items stemming with “I am confident that I can regularly practice physical or sporting activity when …”. The items depict different situations where safe and healthy behaviors must be performed in sports facilities (i.e., “I must avoid using the locker room” or “I don’t have enough time to follow the safety procedures”). The complete list of the original items is reported in the Supplementary Material. All items are rated on a 10-point Likert scale ranging from 1 “not at all confident” to 10 “fully confident.” In a pilot study conducted by the research team through a focus group, the RSES-SR showed good content validity. In our study, the Cronbach’s alpha was 0.88.

### Data Analysis

We used SPSS software Version 25.0 (SPSS Inc., Chicago, IL, United States) for all statistical analyses. The data were initially checked for multivariate outliers and normal distribution through the Kolmogorov–Smirnov test, with the Lilliefors correction. A MANOVA was used to assess if gender (male vs. female), age (younger vs. older), and environment (indoor vs. outdoor) affect sleep, regulatory self-efficacy, optimism, mood, and mental toughness. Effect sizes were calculated using partial eta square ($\eta_{\text{p}}^{2}$) (Lakens, 2013), with 0.01, 0.06, and 0.14 considered small, medium, and large effects, respectively (Cohen, 1988). Significance was set at *p* < 0.05.

## Results

Table 1 displays the demographic and descriptive characteristics of the sample.

**FIGURE 1:**
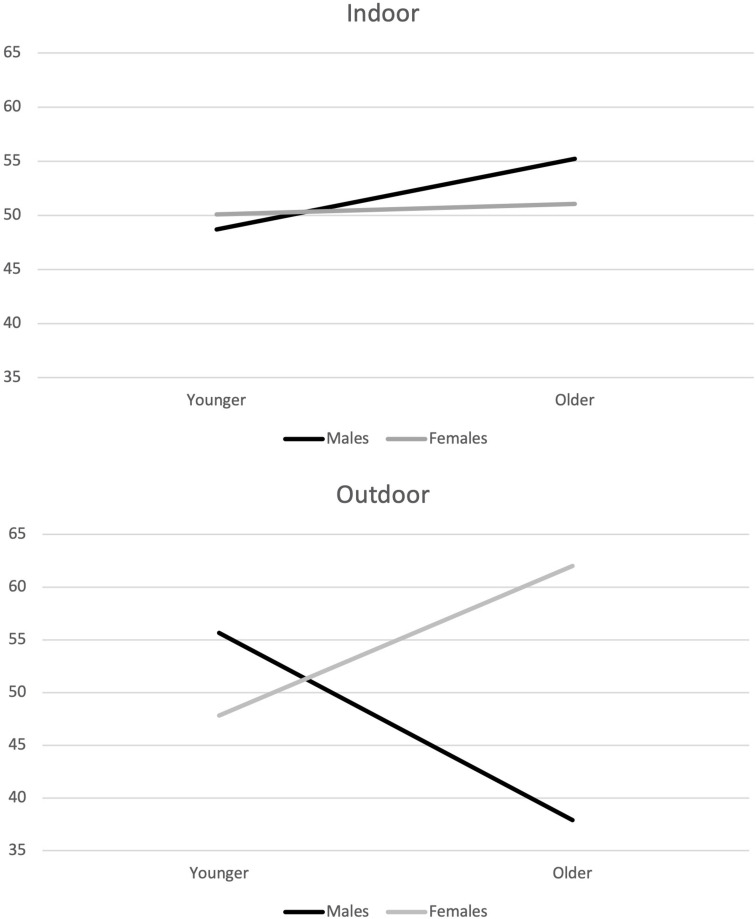
Mean differences in regulatory self-efficacy, based on environment, gender, and age.

About the regulatory self-efficacy, a statistically significant interaction between gender, age, and environment emerged [*F*_(1,321)_ = 8.87, *p* = 0.003] (Figure 1).

**FIGURE 2:**
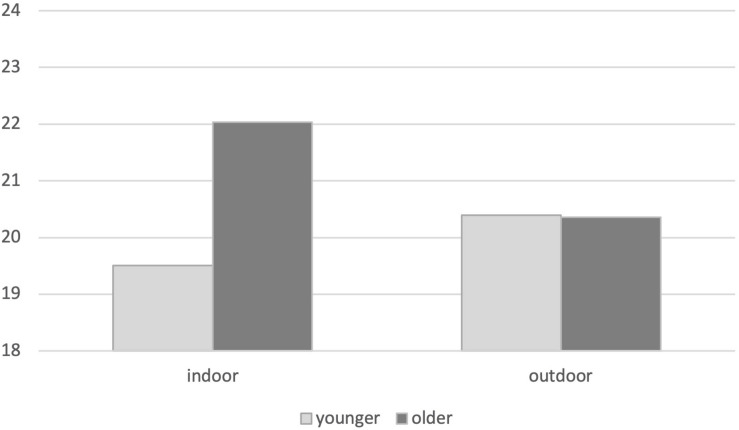
Mean differences in optimism and positive expectations for the future, based on environment and age.

In outdoor physical activities, younger females showed less regulatory self-efficacy than older females, while younger males showed more regulatory self-efficacy than older males. In indoor physical activities, both genders showed similar scores of regulatory self-efficacy related to age, but the patterns between males and females were slightly inverted. A statistically significant interaction between environment and age emerged in optimism and positive expectations for future [*F*_(1,321)_ = 5.74, *p* = 0.017] (Figure 2).

Older individuals practicing indoor physical activities showed more optimism and positive expectations for the future, compared to younger ones. No age differences in life orientation emerged in individuals practicing outdoor physical activities.

Moreover, main statistically significant effects were found only for age in insomnia, mental toughness, and mood states (Table 2).

**TABLE 2:** Main effects of age (estimated means and standard error).

**Psychological variables**	**Younger**	**Older**	***p-*value**	**Effect size**
Insomnia	7.37 ± 0.32	5.92 ± 0.52	**0.018**	0.017
Life orientation	19.92 ± 0.36	21.22 ± 0.59	0.754	0.000
Mental toughness	29.65 ± 0.42	33.47 ± 0.69	**0.000**	0.065
Mood states	55.28 ± 1.03	47.22 ± 1.70	**0.000**	0.048
Regulatory self-efficacy	50.55 ± 1.63	51.54 ± 2.69	0.062	0.011

*The bold font indicates the significance of the result.*

Younger adults, compared to older ones, showed more insomnia and less mental toughness. In mood states, younger ones showed more anger, confusion, depression, fatigue, tension, and less vigor (Table 3).

**TABLE 3:** Mean differences (and standard deviation) in mood states by age.

**Mood states**	**Younger**	**Older**	***p-*value**	**Effect size**
Anger	8.08 ± 3.56	6.96 ± 2.96	**0.003**	0.026
Confusion	8.78 ± 3.82	6.72 ± 3.08	**0.000**	0.075
Depression	7.05 ± 3.50	5.79 ± 2.50	**0.000**	0.037
Fatigue	9.08 ± 3.75	7.09 ± 2.75	**0.000**	0.076
Tension	9.47 ± 4.17	7.51 ± 3.20	**0.001**	0.059
Vigor	12.57 ± 3.08	13.65 ± 2.65	**0.001**	0.032

*The bold font indicates the significance of the result.*

However, all statistically significant effects appeared as a small size, excluding the impact of age in mental toughness, confusion, and fatigues that showed a medium effect size.

## Discussion

The main aim of our study was to evaluate the contribution of different psychological variables to the rebooting of sports and physical activities. To achieve this goal, gender, age, and environment were tested separately, and in their interactions, to assess their effects on insomnia, regulatory self-efficacy, optimism, mood states, and mental toughness. The results of our study partially confirm the initial hypotheses.

Regulatory self-efficacy seems to be a useful predictor of the involvement in sports and physical activities in the rebooting phase. However, its contribution appears to act differently among males and females, in relation to age and to the environment where people regularly performed physical activities. In outdoor physical activities, younger females showed lower regulatory self-efficacy than older females, while younger males showed higher self-efficacy than older males. These differences are reduced and inverted in indoor physical activities. Different explanations could be advanced about these results, based on motivational and situational variables. Males practice physical activity mainly for social and competitive reasons and prefer to practice sports, outdoor and indoor physical activities in public places like the gym and fitness clubs. Females are more inclined to exercise in home-setting, practicing aerobics, yoga dancing, Pilates or circuits with planks, squats, and jumping jacks (Maugeri et al., 2020). Younger females, compared to older ones, might have considered outdoor physical activities and public spaces as unsafe places to practice sports and physical activity during the rebooting phase. Indeed, at the beginning of the resumption phase, even though outdoor physical activities were allowed, it was necessary to avoid gatherings, keep social distances, and use personal protective equipment like masks and gloves. These safety measures may have heightened the young women’s sense of personal insecurity in open environments. On the indoor physical activities, older people showed more optimism and positive expectations than younger people, independently from gender. This result can be explained by the different experience related to age, but also through a higher fear of contagion, which affected the younger people than older people, during the Italian lockdown (Casagrande et al., 2020; Moccia et al., 2020). In the rebooting phase, indoor physical activities required more precautions than outdoor physical activities because of the interdiction of showers or access to locker rooms to limited numbers of people at the same time. These new habits may have reduced the positive expectations of younger people who tend to be more inclined to share experiences, clothes, and spaces than older people.

Contrary to our hypothesis, only age affected most of the psychological variables considered. Younger adults showed more sleep disturbances, confusion, depression, anger, and fatigue, and less vigor and mental toughness than older people. While these differences were expected and confirmed by previous findings (Forte et al., 2020; Somma et al., 2020), we have to notice the absence of gender differences repeatedly found in previous studies conducted in Italy or Spain in the confinement context (López-Bueno et al., 2020a,b; Mazza et al., 2020; Rossi et al., 2020). Sports and physical activities can reduce gender gap also in reported symptoms other than in positive functioning (Health, 2019).

The present research has a few limitations: our exploratory study, based on a self-administered web survey, restricted the participation to the survey only to a convenience sample of people equipped with an Internet connection (Couper, 2000). Furthermore, self-report questionnaires evaluate subjective characteristics and, as is well known, can be biased from social desirability and acquiescence (Meleddu and Guicciardi, 1998). However, the anonymity and the short duration of the survey may have limited these biases. These limitations notwithstanding, to our knowledge, this is the first study investigating the contribution of psychological variables just at the resumption of sports and physical activity in Italy, after a prolonged lockdown, lasting about 3 months.

COVID-19 is not only a novel outbreak circulating worldwide but represents one of the most extensive and most lasting natural experiments conducted to date (Petticrew et al., 2005). It affected the lives of thousands in profound ways and is driving multiple changes in life habits, as wearing or not a face mask or complying with tracing strategies (Jetten et al., 2020). We focused on change in sports and physical activity’s practices and in the psychological variables that can predict a better fit to the new routines. The rebooting phase made it clear that the barriers to keep an active lifestyle were raised. Activities previously experienced as salutary, for example, those conducted in natural environments, now raise concerns and fears, especially for younger women. Indoor physical activities often practiced to increase friendships and sociability have become a cause for apprehension, especially by the younger people, males and females, who have grasped a reason for pessimism in the limitations of the spaces of activity, in the regulation of accesses, and the prohibition of exchanges of equipment. It is too early to understand whether these reactions are temporary or permanent, but at present, young people, as a whole, seem to be more exposed to the unexpected effects of the restrictions implemented to limit the spread of the virus during the rebooting of sports and physical activities. These results confirm the needs to harness psychology as a critical element to understand and incentive health and physical activity behaviors in a changing world. Considering these differences can help to support new habit formation and to enact more appropriate actions to practice sports and physical activities when living at the time of Coronavirus.

## Data Availability Statement

The raw data supporting the conclusions of this article will be made available by the authors, without undue reservation, to any qualified researcher.

## Ethics Statement

The studies involving human participants were reviewed and approved by the Ethics Committee of the University of Cagliari. The patients/participants provided their written informed consent to participate in this study.

## Author Contributions

MG and RP were responsible for the study, design, methodology, data analysis, and drafting and editing of manuscript. Both authors have read and agreed to the published version of the manuscript.

## Conflict of Interest

The authors declare that the research was conducted in the absence of any commercial or financial relationships that could be construed as a potential conflict of interest.
