# Supplementary material for: The Rebooting in Sports and Physical Activities After COVID-19 Italian Lockdown: An Exploratory Study
Source: Front Psychol. 2020 Nov 25;11:607233. doi: 10.3389/fpsyg.2020.607233 (PMC7723834; doi:10.3389/fpsyg.2020.607233)
Supplement: Supplementary file 1 [file Table_1.DOCX]

Supplementary Material

Regulatory Self-Efficacy Scale for Sport Rebooting (RSE-SR): list of items

1. il luogo in cui pratico sport/attività fisica è particolarmente affollato;
2. posso evitare di entrare negli spogliatoi;
3. devo condividere gli attrezzi con altre persone;
4. devo lasciare i miei effetti personali incustoditi nello spogliatoio;
5. qualche persona presente non rispetta le distanze di sicurezza;
6. sono presenti cibo e bevande non consumate negli spazi comuni;
7. non c’è nessuno che fa rispettare le disposizioni di sicurezza;
8. posso fare a meno di utilizzare i servizi igienici;
9. qualche persona presente non indossa i sistemi di protezione individuale (mascherine/guanti);
10. non ho sufficiente tempo per seguire le procedure di sicurezza;
11. l’ambiente non permette un ricircolo dell’aria.
